# Comparative Transcriptome Analysis of *Gossypium hirsutum* Mutant (*xin w 139*) and Wild-Type (Xin W 139) Plants During Seed Embryo Development

**DOI:** 10.3390/genes15111408

**Published:** 2024-10-30

**Authors:** Jieyin Zhao, Chunping Li, Yanlong Yang, Jun Ma, Chengxia Lai, Paerhati Maimaiti, Liwen Tian

**Affiliations:** 1 Institute of Cash Crops, Xinjiang Academy of Agricultural Sciences, Urumqi 830091, China; cottonzjy@126.com (J.Z.); chunpin96@126.com (C.L.); yangyl0629@163.com (Y.Y.); xj.majun@163.com (J.M.); lchxia2001@163.com (C.L.); 2 College of Agriculture, Xinjiang Agricultural University, 311 Nongda East Road, Urumqi 830052, China; 3 China National Cotton R&D Center, Urumqi 830091, China

**Keywords:** *Gossypium hirsutum*, mutants, seed embryo development, RNA-seq, candidate genes

## Abstract

Background: Cotton seeds are the main byproduct of cotton crops. The phenomenon of plants failing to develop mature and full seeds is called seed embryo abortion, which leads to a decrease in seed yield and potentially causes economic losses. Methods: We report a phenotypic evaluation of seed embryos from *G. hirsutum* mutant (*xin w 139*) and wild-type (Xin W 139) lines and a comparative RNA-seq study at four developmental stages. Results: The field results from two years showed that the sterility rate and malformation rate of *xin w 139* were significantly lower than those of Xin W 139, and the RNA-seq data revealed that the differences in the development of the seed embryos of the two lines mainly occurred after 20 days post anthesis (DPA). Differential analysis revealed a total of 29,151 differentially expressed genes (DEGs), including 2696 transcription factors (TFs), between the two lines, in which the fatty acid and glucose metabolism-related pathways were significantly enriched. These DEGs were divided into 8 clusters, with the Kyoto Encyclopedia of Genes and Genomes (KEGG) pathways of each cluster being annotated. Furthermore, a gene regulatory network was built using weighted correlation network analysis (WGCNA), revealing 9 key genes that play crucial roles in shaping the developmental disparities of seed embryos between the two lines, among which 3 are TFs. Conclusions: These findings offer a foundational framework for comprehending the molecular mechanisms underlying cottonseed embryo development, as well as presenting novel genetic reservoirs for further investigations into cottonseed embryo development.

## 1. Introduction

Cotton, being the primary fiber and oil crop globally, generates cottonseed as its key byproduct, comprising around 60% of the overall cotton yield. The oil content in cottonseed ranges between 15.0% and 48.7% [1]. However, cotton breeders have long focused primarily on improving fiber quality, yield, and disease resistance in cotton plants [2,3]. Although there have been some studies on the development of cottonseed embryos, these studies have been limited to the analysis of mature cotton [4]. Mature cottonseed contains many nutrients, which vary according to the different cotton species and cultivars, and the average nutrient content component breakdown is roughly as follows: 23% carbohydrate, 22% oil, 20% protein, 20% crude fiber, 10% moisture, and 5% ash [1]. Cottonseed husks account for approximately 40% of the weight of cottonseeds, and their composition is mainly crude fiber and carbohydrates [5]. The seed accounts for approximately 60% of the weight of cottonseed, and its components are mainly oil and protein, with oil accounting for 35~46%, protein accounting for 30~35%, and carbohydrates accounting for approximately 15%. In addition, cottonseed also contains a certain amount of gossypol and ash [5].

The seed is the beginning of the life of the offspring of an adult plant; it is also the result of the sexual reproduction of the parents and is the product of the reproductive growth process. In the early stages of development, the egg cell and sperm combine to develop into a zygote, which then undergoes cell division, organ formation, and finally maturation [6]. Cottonseeds are mainly composed of two parts, the seed coat and the seed embryo, and there is also a milky white film, which is tightly wrapped around the seed embryo, where the endosperm resides [7]. The seed embryo is composed of four parts: cotyledon, radicle, germ, and hypocotyl [8]. The outside of the embryo is surrounded by a milky white film, which is the residue of the bead core and endosperm tissue. Most of the space of the seed embryo is occupied by two cotyledons [9]. The large and small cotyledons are close together, folded in a tortuous shape, and are born on the hypocotyl. The hypocotyl is connected to the radicle, and the upper part is the germ. In general, the germs of mature seeds differentiate into two true leaf primordia, while semimature cottonseeds differentiate into only one true leaf primordium [10]. Each part of the seed embryo has black or brown pigment glands, except for the radicles, which are mainly gossypol. Low-phenol or nonphenolic cotton has very few or no glands on the seeds [11]. The morphogenesis of cottonseed embryos is divided into tissue differentiation, cell enlargement, and maturation dehydration, and these three stages overlap [12]. Because the abortion of seeds leads to a lack of the full appearance of harvested seeds, the seed deformity rate can be used as an important reference index. This phenomenon is the result of the joint action of many factors during the development process. Often, individuals with poor seed density are prone to abortion, and their seed fat content also increases [13].

The transcriptome encapsulates all RNA transcripts within a cell or tissue, showcasing the genes expressed across various life stages, tissue varieties, physiological states, and environmental contexts. The widespread adoption of RNA-seq, facilitated by high-throughput technology, has made it a versatile tool for delving into the molecular underpinnings of biological traits across diverse research domains [14]. Using RNA-seq analysis on 31 samples of early B73 kernel development (including embryo sacs) within 6 days post-double fertilization, a sum of 22,790 genes (comprising 1415 transcription factors (TFs)) were observed to be expressed in a minimum of one sample. Among these, there were a total of 1093 grain-specific genes identified, with 110 being transcription factors. Surprisingly, 654 of these grain-specific genes were previously uncharacterized [15]. Recently, 598 peel-specific genes, including 75 TFs, were identified based on RNA-seq data from 21 samples of maize B73 seed coat development from 5 days before fertilization to 32 days after fertilization. These data were analyzed with nonseed, embryonic, endosperm, and nuclear cell RNA-seq, and the results indicated that cell wall-related genes play a key role in the construction of maize seed coat structures [16]. Despite the insights gained from the RNA-seq analysis of critical regulatory networks and genes associated with cottonseed embryo development and stress resistance, which offers valuable leads for enhancing cotton quality and resilience to stress, there are few investigations into cottonseed embryo development in the literature [17,18]. To remedy this, we conducted an assessment of 2-year-old sterile seeds, deformed seeds, and regular seeds from *G. hirsutum* Xin W 139 and mutant *xin w 139*. Subsequently, RNA-seq analyses were performed across the four stages of seed embryo development. By comparing the transcriptomes of mutants and wild-type plants during seed embryo development, it is possible to understand the changes in gene expression during seed embryo development and identify key regulatory genes and pathways. These findings establish a solid foundation for comprehensively unraveling the molecular pathways involved in cottonseed embryo development and introduce novel genetic reservoirs for further exploration in this domain. This study is highly important for improving seed yield and quality and improving seed utilization value.

## 2. Materials and Methods

### 2.1. Plant Materials

**Figure 1:**
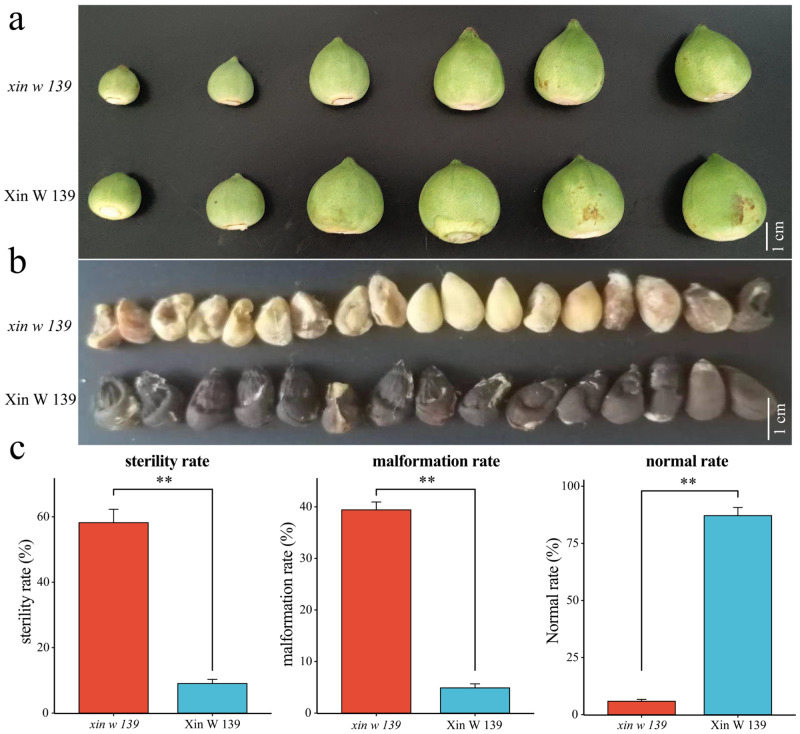
Phenotype and sterility rate, malformation rate and normal rate of cotton bolls and seeds of Xin W 139 and *xin w 139*. (**a**) The phenotypes of the Xin W 139 and *xin w 139* cotton bolls; bar = 1 cm. (**b**) The phenotype of the seeds after maturity of Xin W 139 and *xin w 139*, bar = 1 cm. (**c**) Statistical analysis of the sterility rate, malformation rate, and normal rate of Xin W 139 and *xin w 139*. Significant differences were tested by *t* tests using one-way ANOVA (** *p* < 0.01).

In the present study, a previously identified fibrous immature mutant (*xin w 139*) and its wild type (Xin W 139) were used. In 2022, statistics on the sterility, malformation rate, and normal rate of Xin W 139 and *xin w 139* seeds were carried out in Manas County, Changji city, Xinjiang, and in Toutai township, Wusu city, Xinjiang, in 2023 (no fewer than 5000 seeds were counted in each line; the phenotypes of the cotton bolls and seeds are shown in Figure 1). During the field experiment, three rows of each line were cultivated with each plot stretching over 8 m in length. The row and plant spacings were set at 0.70 m and 0.10 m, respectively, and standard management practices consistent with the local conventional fields were implemented. Identifying cotton bolls at the onset of flowering as 0 days post anthesis (DPA), samples were collected at 11 a.m. on days 15, 20, 25, and 30 DPA. Within 1 min of the cotton boll harvest, the cotton shell was promptly removed, followed by the swift extraction of the seed embryo. Subsequently, the seed coat was carefully removed using tweezers, and the seed embryo was rapidly frozen in liquid nitrogen. Three biological replicates were obtained from each sample.

### 2.2. RNA Extraction, cDNA Library Preparation, and Sequencing

The TRIzol method was employed for the RNA extraction, with an RNA integrity assessment conducted through 1% agarose gel electrophoresis. Subsequently, the total RNA samples were shipped to Maiwei Metabolism Company (Wuhan, China) for sequencing. The isolated RNA underwent fragmentation in a PCR plate featuring a magnetic plate holder. Following this, mRNA fragments underwent reverse transcription into cDNA utilizing Superscript II and random primers (Invitrogen, Carlsbad, CA, USA). The fastp software (version 0.23.4) was employed to eliminate adapter sequences, filter out low-quality sequences with over 5% N sequences, and more, in order to procure refined reads suitable for further analysis [19]. The genome from G. hirsutum TM-1, accessible at https://www.cottongen.org/species/Gossypium_hirsutum/ZJU-AD1_v2.1 (accessed on 6 July 2023), served as the reference for the analysis. The alignment of reads was executed using HISAT2, while String Tie was harnessed for quantifying the reads within the alignment [20,21,22].

### 2.3. Analysis of DEGs

The fragments per kilobase of exon per million fragments mapped (FPKM) quantifies the gene expression by calculating the number of reads per million mapped reads that align to exons, normalized to the length of the exon in per thousand bases. This FPKM method is utilized for assessing gene expression levels. Utilizing the DESeq2 software (version 1.44), the fold changes in the differential gene expression were computed based on the raw read count data of the genes. Subsequently, the false discovery rate (FDR) was ascertained by adjusting the *p*-value through multiple hypothesis testing utilizing the Benjamini–Hochberg method [23]. The DEGs were screened based on criteria that included an FDR ≤ 0.01 and a |log2-fold change| ≥ 1 [24]. The DEGs were annotated based on the Kyoto Encyclopedia of Genes and Genomes (KEGG) database (http://www.genome.jp/kegg/, accessed on 15 July 2023). The DEG genome sequences were submitted to the PlantTFDB (http://planttfdb.cbi.pku.edu.cn/, accessed on 15 July 2023) for transcription factor prediction.

### 2.4. Construction of Coexpression Networks

The gene expression patterns of the DEGs were assessed for co-expression using the R language WGCNA package (version 1.73), involving dynamic branch cutting. It is recommended that the weighting coefficient in this process aligns closely with 0.8 [25]. The network construction for the gene co-expression modules was carried out utilizing the automated network builder Blockwise Modules. These modules varied in gene count, and those modules showing a similarity of 0.75 were merged according to the standards of minModuleSize = 30 and Merge Cut Height = 0.25. Subsequently, the correlation coefficient between the characteristic vector module eigengene (ME) and the distinct time points during the seed embryos development in the two lines were computed. The co-expression networks were visualized via Cytoscape (version 3.9.0) software [26].

### 2.5. qRT-PCR

The total RNA extraction process was conducted utilizing the RNAprep Pure Polysaccharide Polyphenol Plant Total RNA Isolation Kit (Tiangen, Sichuan, China). Subsequently, the concentration of each RNA sample was assessed through a NanoDrop 2000 spectrophotometer (Thermo Fisher Scientific, Waltham, MA, USA). cDNA was obtained by RNA reverse transcription using the M-MLV RTase cDNA Synthesis Kit (TaKaRa, Shiga, Japan). qRT-PCR analysis was performed using a Bio-Rad CFX96 Real-time System device (Mannheim Roche Diagnostics GmbH, Mannheim, Germany) and 20 μL of iTaq Universal SYBR Green Supermix (Takara Bio, Inc., Shiga, Japan). The reaction program was as follows: predenaturation at 95 °C for 30 s, denaturation at 95 °C for 5 s, annealing at 60 °C for 5 s, and extension at 72 °C for 34 s for 40 cycles. Relative quantitative analysis was performed by the 2^−ΔΔCt^ method [27]. The internal reference gene was *GhUBQ7*, and there were three biological replicates for each program. All the primers used in this study are shown in Table S1.

## 3. Results

### 3.1. Phenotypic Identification of Xin W 139 and xin w 139

The abortion and malformation of seeds are key indicators for evaluating the attainment of seed maturity, so we collected information on the sterility, malformation, and normality rates of seeds of Xin W 139 and *xin w 139* in 2022 and 2023, respectively. The sterility rate and deformity rate of the *xin w 139* seeds were significantly greater than those of the Xin W 139 seeds (Figure 1c). This is mainly caused by the immaturity of the seed embryo during seed development. For a deeper exploration into the molecular mechanisms and candidate genes driving the developmental distinctions between Xin W 139 and *xin w 139* seed embryos, RNA-seq analysis was undertaken on seed embryos samples obtained from the four developmental stages (15 DAP, 20 DAP, 25 DAP, and 30 DAP) of the two lines.

### 3.2. RNA-seq Analysis

**Figure 2:**
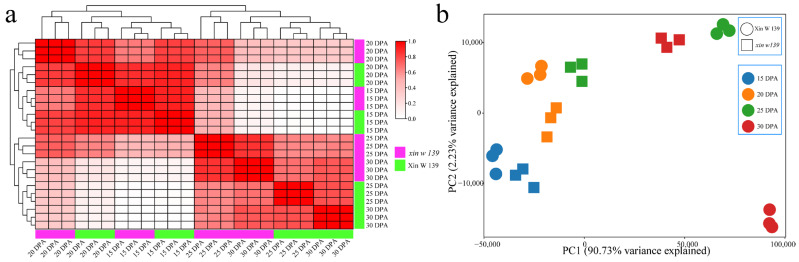
Correlation and PCA of RNA-seq samples at four stages of embryonic development in Xin W 139 and *xin w 139*. (**a**) Correlation line and cluster analysis of RNA-seq samples from 24 cotton embryo development samples; the red for the correlation, the purple for *xin w 139*, and the green for Xin W 139. (**b**) PCA of 24 RNA-seq-identified cotton embryo development samples; the different colors represent different stages of mesodermal development, with a circle representing Xin W 139 and a square representing *xin w 139*.

The RNA-seq analysis involved 24 samples encompassing the two lines and four developmental stages of seed embryos, yielding a total of 188.02 Gb of clean data. Each sample met the criteria of having clean data exceeding 7.11 Gb, having a Q30 base percentage exceeding 92.46%, having a GC content exceeding 43.54%, and exhibiting alignment rates ranging from 98.11% to 98.47%, with an average of 98.31% (Table S2). First, correlation cluster analysis of the samples was carried out, and the correlation between the samples of the same biological replicate was greater than 0.97, indicating that the transcriptome data were reliable and reproducible (Figure 2a). To elucidate the transcriptome dynamics of the cottonseed embryo development, we performed principal component analysis (PCA) on samples from the two lines and four development time points. The correlation between the two lines was greatest at 15 days post anthesis (DPA), the PCA clustering was the most recent, the correlation decreased with the development of the seed embryo, and the clustering of the PCA was more distant (Figure 2b). Among these samples, those from which the *xin w 139* seed embryo developed at 30 DPA were clustered with the 25 DPA samples from Xin W 139, suggesting that the main reason for the immaturity of the *xin w 139* seeds may be their developmental differences after 20 DPA.

### 3.3. Differential Expression Analysis within Lines

**Figure 3:**
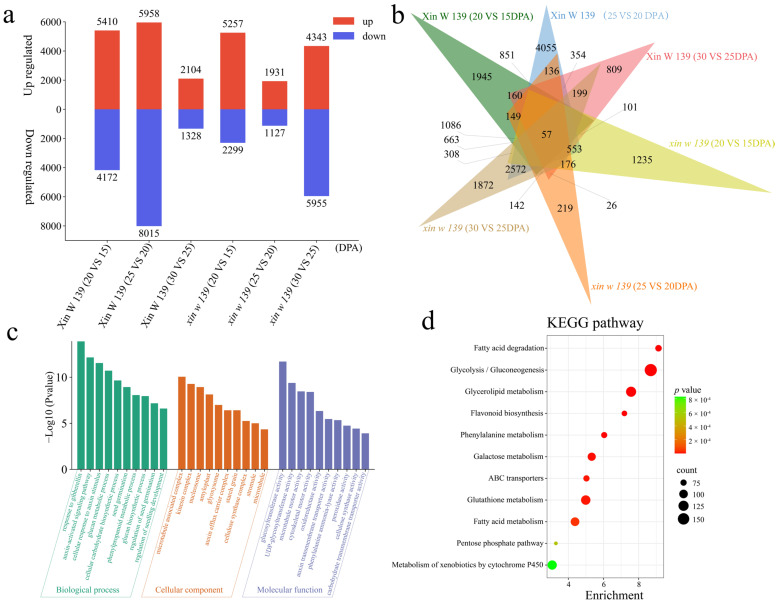
Differential expression analysis and enrichment analysis of DEGs in Xin W 139 and *xin w 139* developmental stages in embryos of the same line. (**a**) The number of DEGs whose expression was upregulated or downregulated during different developmental stages in embryos of the same line. (**b**) Venn diagram of DEGs at different developmental stages in embryos of the same line; the overlapping regions are common DEGs. (**c**) GO enrichment analysis of DEGs during different developmental stages of embryos of the same line; the higher the column, the smaller the *p*-value. (**d**) KEGG pathway enrichment analysis of DEGs during different developmental stages of embryos of the same line. The size of the dot represents the number of genes in the pathway, a larger dot represents a greater number of genes, and the color of the dot represents the size of the *p*-value; a redder dot represents a smaller *p*-value, and a greener dot represents a larger *p*-value.

To study the transcriptional differences between the two lines at different stages of the seed embryo development, DEGs were identified at each stage of seed embryo development in the same line, and 57 common DEGs were identified between the two lines (Figure 3a,b). In Xin W 139, there were 9582 DEGs between 20 DPA and 15 DPA, of which 5410 DEGs were upregulated and 4172 DEGs were downregulated, including 1945 unique DEGs. There were 13,973 DEGs between 25 DPA and 20 DPA, of which 5958 DEGs were upregulated and 8015 DEGs were downregulated, including 4055 unique DEGs. There were 3432 DEGs between 30 DPA and 25 DPA, of which 2104 DEGs were upregulated and 1328 DEGs were downregulated, including 809 unique DEGs. In *xin w 139*, there were 7556 DEGs between 20 DPA and 15 DPA, of which 5257 DEGs were upregulated and 2299 DEGs were downregulated, including 1235 unique DEGs. There were 3058 DEGs between 25 DPA and 20 DPA, of which 1931 DEGs were upregulated and 1127 DEGs were downregulated, including 219 unique DEGs. There were 10,298 DEGs between 30 DPA and 25 DPA, of which 4343 DEGs were upregulated and 5955 DEGs were downregulated, including 1872 unique DEGs. Gene Ontology (GO) enrichment analysis of 24,114 DEGs from the same line at different times revealed significant annotations related to the response to gibberellin, the auxin-activated signaling pathway, the cellular response to auxin stimulus, the glucan metabolic process, the cellular carbohydrate biosynthetic process, seed germination, the phenylpropanoid metabolic process, the glucan biosynthetic process, the regulation of seed germination, and the regulation of seedling development (Figure 3c). The KEGG pathways involved were mainly fatty acid degradation, glycolysis/gluconeogenesis, glycerolipid metabolism, flavonoid biosynthesis, phenylalanine metabolism, galactose metabolism, ABC transporters, glutathione metabolism, fatty acid metabolism, the pentose phosphate pathway, and metabolism of xenobiotics by cytochrome P450.

### 3.4. Expression Analysis of Differences between Lines

**Figure 4:**
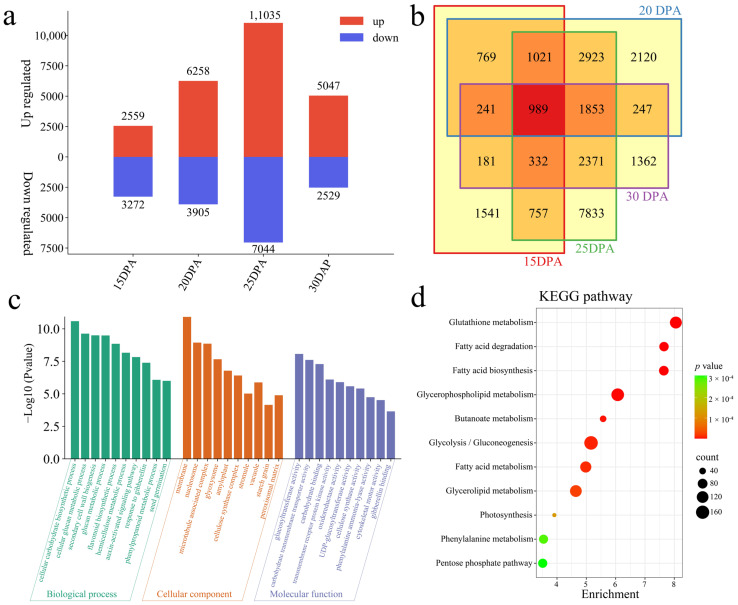
Differential expression analysis and DEGs enrichment analysis among embryonic the same stages between lines of Xin W 139 and *xin w 139* embryos. (**a**) The number of DEGs whose expression increased or decreased during the same period between lines. (**b**) Venn diagram of DEGs between lines; the overlapping regions are common DEGs. (**c**) GO enrichment analysis of DEGs between lines; the higher the column, the smaller the *p*-value. (**d**) KEGG pathway enrichment analysis of DEGs between lines. The size of the dot represents the number of genes in the pathway, a larger dot represents a greater the number of genes, and the color of the dot represents the size of the *p-*value; a redder dot represents a smaller *p-*value, and a greener dot represents a larger *p-*value.

By identifying the DEGs of the Xin W 139 and *xin w 139* seed embryos at the same stage of development, 989 common DEGs were identified (Figure 4a,b). At 15 DPA, there were 5831 DEGs, of which 2559 DEGs were upregulated, 3272 DEGs were downregulated, and 1541 were unique DEGs. At 20 DPA, there were 10,163 DEGs, of which 6258 DEGs were upregulated, 3905 DEGs were downregulated, and 2120 were unique DEGs. At 25 DPA, there were 18,079 DEGs, with 11,035 DEGs upregulated, 7044 DEGs downregulated, and 7833 unique DEGs. At 30 DPA, there were 7576 DEGs, of which 5047 DEGs were upregulated, 2529 DEGs were downregulated, and 1362 were unique DEGs. Through the GO enrichment analysis of 24,540 DEGs between the lines, it was found that the main annotations were the cellular carbohydrate biosynthetic process, cellular glucan metabolic process, secondary cell wall biogenesis, glucan metabolic process, flavonoid biosynthetic process, hemicellulose metabolic process, auxin-activated signaling pathway, response to gibberellin, phenylpropanoid metabolic process, and seed germination biological process (Figure 4c). The KEGG pathways involved were mainly glutathione metabolism, fatty acid degradation, fatty acid biosynthesis, glycerophospholipid metabolism, butanoate metabolism, glycolysis/gluconeogenesis, fatty acid metabolism, glycerolipid metabolism, photosynthesis, phenylalanine metabolism, and the pentose phosphate pathway (Figure 4d).

### 3.5. Cluster Analysis of DEGs

**Figure 5:**
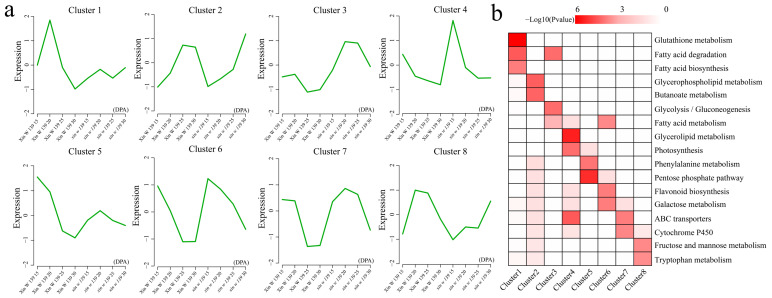
k-means clustering analysis of all DEGs within and between materials and KEGG enrichment analysis for each cluster. (**a**) DEGs k-means clustered line chart, with clusters with inner squares and decreases to flatten out as optimal clusters. (**b**) KEGG pathway annotated heatmap with different clusters of k-means; the darker the red, the smaller the *p*-value.

A total of eight statistically significant clusters were identified for 29,151 DEGs using k-means clustering, and KEGG enrichment analysis (Figure 5a,b) was performed for each individual cluster. The Cluster 1 expression trend was consistent in both lines but was greatest at 20 DPA in Xin W 139, which was significantly associated with the glutathione metabolism, fatty acid degradation, and fatty acid biosynthesis pathways. In Cluster 2, the expression in Xin W 139 first increased at 25 DPA to a maximum, and the expression in *xin w 139* increased at 30 DPA to a maximum, which was significantly related to the glycerophospholipid metabolism and butanoate metabolism pathways. The expression of Cluster 3 in Xin W 139 first increased slightly and then reached a minimum at 25 DPA, and the expression in *xin w 139* decreased slightly at 25 DPA and reached a minimum at 30 DPA. These genes were significantly annotated in the fatty acid degradation, glycolysis/gluconeogenesis, and fatty acid metabolism pathways. The expression of Cluster 4 decreased in both lines, reached a minimum at 30 DPA, and was significantly associated with the glycerolipid metabolism, photosynthesis, and ABC transporter pathways. The decrease in the Cluster 5 expression in Xin W 139 reached a minimum at 30 DPA, and the expression in *xin w 139* first increased and then reached a maximum at 20 DPA, which was significantly associated with the phenylalanine metabolism and pentose phosphate pathways. The expression of Cluster 6 decreased in both lines, and at 25 DPA and 30 DPA in Xin W 139, it was significantly related to the fatty acid metabolism, flavonoid biosynthesis, and galactose metabolism pathways. The Cluster 7 expression decreased in Xin W 139 and then decreased in *xin w 139*, which was significantly related to the ABC transporter and cytochrome P450 pathways. The expression of Cluster 8 in Xin W 139 first increased and then decreased with a maximum at 20 DPA, and the expression of in *xin w 139* always increased to a maximum at 30 DPA, which was significantly related to the fructose and mannose metabolism and tryptophan metabolism pathways.

### 3.6. TF Expression Analysis

**Figure 6:**
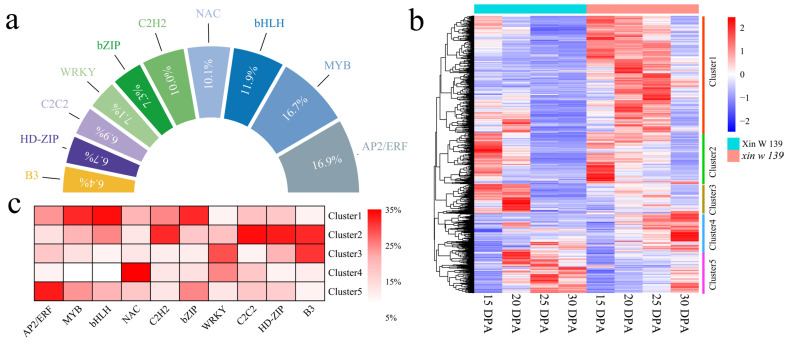
Statistical analysis of all differentially expressed TFs within different stages and between lines same line and heat maps of expression patterns. (**a**) Percentages of genes associated with the top 10 transcription factors. (**b**) Heatmap of the clustering of differentially expressed transcription factors according to gram-means clustering. (**c**) Heatmap of the percentage of transcription factors in different clusters; the darker the red, the higher the percentage.

A total of 2696 differentially expressed TFs, mainly AP2/ERF, MYB, bHLH, NAC, C2H2, and bZIP, were identified among the 28,574 DEGs (Figure 6a). A total of five statistically significant clusters (Figure 6b) were identified for the differential 1890 TFs using k-means clustering. Cluster 1 had the highest expression at 15–25 DPA and was detected in *xin w 139*, which mainly included transcription factors such as MYB, bHLH, and bZIP (Figure 6c). After 15 DPA, the expression of the genes in Cluster 2 gradually decreased in both lines, and these genes mainly included genes encoding transcription factors such as C2H2, C2C2, HD-ZIP, and B3. The expression level of Cluster 3 in Xin W 139 showed a decreasing trend and slightly increased in *xin w 139*, which mainly included transcription factors such as WRKY and B3. The Cluster 4 expression increased slightly in both lines and tended to increase in *xin w 139*, mainly related to transcription factors such as NAC and WRKY. The expression of genes in Cluster 5 increased in both lines and increased in Xin W 139, mainly including genes encoding transcription factors such as AP2/ERF, MYB, and bZIP.

### 3.7. WGCNA and Candidate Gene Annotation

**Figure 7:**
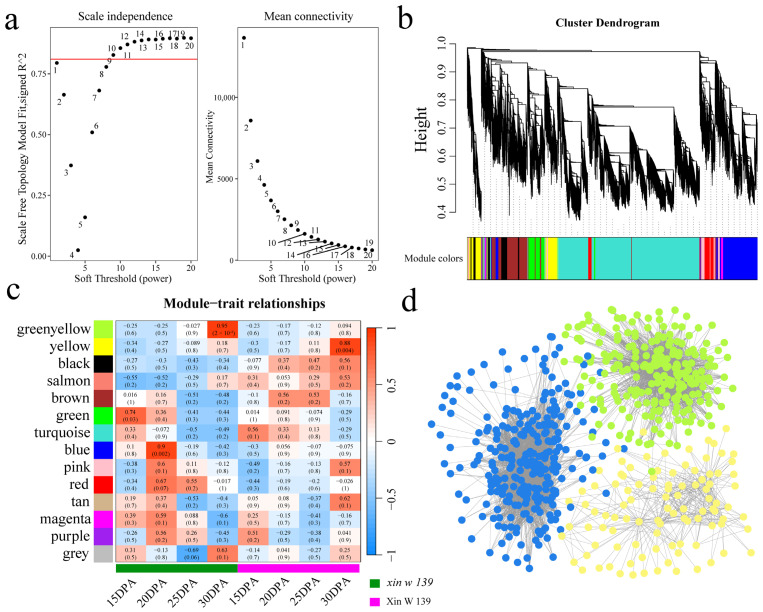
WGCNA of all DEGs within and between lines. (**a**) Scale-free topology fit index as a function of the soft-thresholding power; the red line represents that R^2^ is equal to 0.8. (**b**) Hierarchical clustering tree of 29,151 DEGs based on coexpression network analysis. (**c**) Heatmap of correlations and significance between modules and different periods of seed embryos development. (**d**) Gene coexpression network within the green-yellow, yellow and blue modules.

To construct the cottonseed embryo development gene coexpression network and mine the core genes from it, the expression matrix of 29,151 DEGs was used for WGCNA, and the optimal soft threshold β = 9 was selected (Figure 7a) by the dynamic tree cutting method. A total of 14 different coexpression modules were identified. The largest and smallest modules were the turquoise and dark red modules, which contained 3,355 and 49 genes, respectively (Figure 7b). According to the correlation results between the module and the two lines at the different stages of seed embryo development, the green-yellow module was significantly highly correlated with *xin w 139* 30 DPA (r = 0.95, *p* < 0.01), the yellow module was significantly highly correlated with Xin W 139 30 DPA (r = 0.88, *p* < 0.01), and the blue module was significantly highly correlated with *xin w 139* 20 DPA (r = 0.90, *p* < 0.01) (Figure 7c). Cytoscape 3.9.0 software was used to construct the gene interaction networks of these key modules (green-yellow, yellow, and blue), and the MCC algorithm of the CytoHubba (version 0.1) plug-in in the software was used to screen the top three core genes in the interaction network as candidate genes. For each module, the three genes with the highest degree of linkage were identified as candidate genes, and nine candidate genes were ultimately identified (Figure 7d). To explain the relationships among the nine candidate genes and the development of the cottonseed embryos, the core genes were aligned to the Arabidopsis genome with BLAST, and the functions of the candidate genes were annotated with the help of homologous Arabidopsis genes (Table 1). Three genes encode transcription factors, namely, *GH_D02G1381* (MADS), *GH_D03G0690* (MYB), and *GH_D10G0604* (C3H). *GH_D02G1381* is involved mainly in the control of flowering time, *GH_D03G0690* is involved mainly in trichome and endosperm development, and *GH_D10G0604* is involved mainly in seed germination and seedling/seed development. *GH_A07G0877*, encoding a UDP-arabinopyranose mutase, is involved mainly in amino sugar and nucleotide sugar metabolism; *GH_D01G1934*, encoding a flavin-containing monooxygenase, is involved mainly in carotenoid biosynthesis; and *GH_D02G0314*, encoding a glutathione-S transferase, is involved mainly in glutathione metabolism. *GH_D06G0373* encodes trehalose 6-phosphate phosphatase, which is mainly involved in starch and sucrose metabolism. *GH_D10G0072* encodes a berberine bridge enzyme-like gene and is involved mainly in phenylpropanoid biosynthesis. *GH_D11G3021*, encoding a NADH/NADPH oxidase, is involved mainly in the MAPK signaling pathway.

### 3.8. qRT-PCR

**Figure 8:**
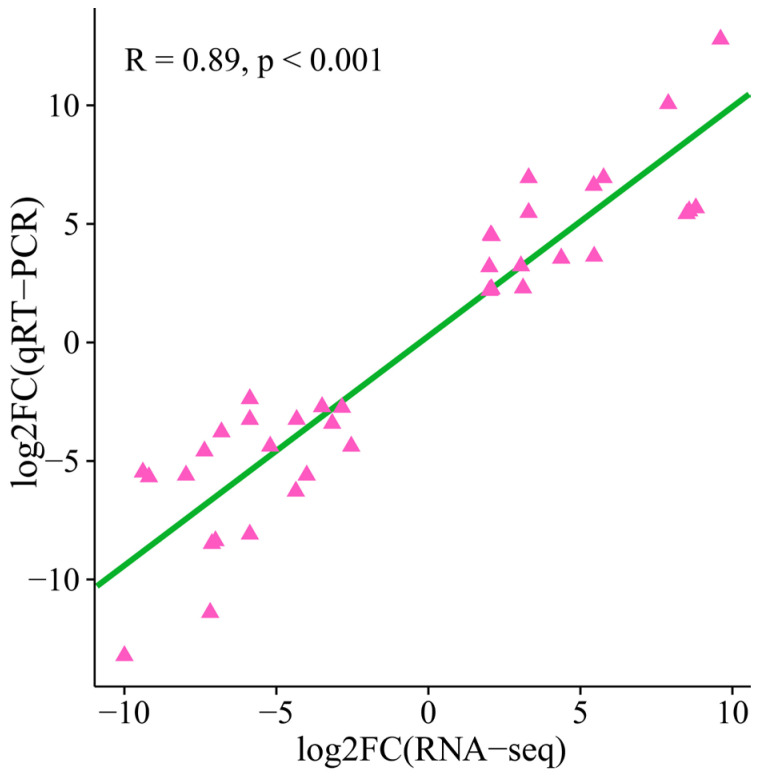
Scatter plot of the correlation between the RNA-seq and qRT-PCR gene expression levels.

In order to validate the precision of the RNA-seq expression profile, nine candidate genes underwent three separate replicates of qRT-PCR. The correlation between the qRT-PCR and RNA-seq fold differences was computed. The outcomes indicated a substantial correlation between the transcriptome data and the qRT-PCR data (R = 0.89, *p* < 0.01), highlighting the reliability of the transcriptome sequencing data (Figure 8).

## 4. Discussion

Seed plants begin their life cycle at seed germination, continue to grow and develop, and transition from vegetative growth to reproductive growth, which is a major turning point in their growth and development process [28]. Embryonic development is an extremely important step in the process of plant growth and development, but not all plants develop normally and smoothly when reproductive offspring are produced [29]. Therefore, the failure to develop mature full seeds during sexual reproduction is called seed embryo abortion [30]. Many plants, such as rice, wheat, *Zea mays*, millet, soybean, peanuts, rape, litchi, and grape, experience embryo abortion, especially some crop seed embryo abortion problems, which can result in serious economic losses [31,32,33]. On the one hand, the problem of embryo abortion is often reduced by a reduction in the fruit setting rate caused by embryo abortion, which seriously restricts breeding [34]. On the other hand, embryo abortion can result in the formation of near-seedless or seedless fruit. This is considered an excellent fruit trait, as seen in seedless grapes, seedless watermelon, and seedless jujubes, which are convenient to eat and are loved by consumers [35]. Therefore, the mechanism of plant seed embryo abortion has been a popular research topic and has been extensively studied in many crops. Here, we performed two consecutive phenotypic evaluations based on previously reported immature mutant (*xin w 139*) and wild-type (Xin W 139) lines and RNA-seq at four time points of seed embryo development. The sterility and deformity rates of *xin w 139* were significantly greater than those of Xin W 139. PCA and cluster analysis of the samples revealed that the Xin W 139 25 DPA sample was closest to the *xin w 139* 30 DPA sample, and the key to the difference between *xin w 139* and Xin W 139 seed embryos may be the developmental difference after 20 DPA.

In plants, oil body proteins can be decomposed by structural proteases. Seed oil body proteins include many proteins, especially in oil crops [36]. The main oil body proteins (similar to oleic acid, calcin protein, and steroids) have been shown to play important roles in lipid accumulation and regulation. Studies have shown that the main function of oil body proteins is to store neutral lipids during seed dormancy [37]. After seed imbibition and germination, neutral lipids decompose and then provide energy and carbon sources to support seedling growth [38]. Moreover, fatty acid accumulation inhibits neutral lipolysis, thus hindering seed development processes [39]. In this study, we found that fatty acid biosynthesis, fatty acid degradation, and fatty acid metabolism pathways were also significantly enriched among the lines or at the different developmental stages of the lines, and many DEGs related to fatty acid pathways were upregulated. Thus, fatty acid accumulation and degradation are crucial for seed development, and fatty acid degradation can promote neutral lipid breakdown, thereby providing nutrient storage and distribution for seed germination. Long-chain acyl-CoA synthetase (ACSL) is a key enzyme involved in the process of fatty acid degradation, and we detected three CSL genes (*GH_D11G0243*, *GH_A01G0241* and *GH_A02G1129*) between and within the lines.

Auxin is involved in regulating the final structure and size of the embryo. Fertilized ovules are enriched in auxin during development, which initiates central cell division and cellularization of the endosperm during endosperm development and affects seed size [40]. In auxin biosynthesis, the tryptophan aminotransferase of *Arabidopsis thaliana* (TAA)/YUCCA (YUC) is important for embryogenesis and organ differentiation after double fertilization, and the *A. thaliana yuc 1*, *yuc 4*, *yuc 10*, and *yuc 11* deficiency mutants fail to form normal hypocotyls and root meristems [41]. We also identified three YUC genes among the DEGs (*GH_A05G0103*, *GH_A07G0283*, and *GH_D08G1408*) in the present study. In rice, *Bg 1-D* is involved in auxin transport and distribution, and overexpression of *Bg 1-D* can significantly increase seed size [42]. PIN-FORMED (PIN) proteins are a class of proteins that regulate the polar transport of auxin. Changes in the structure or conformation of the PIN will affect the orderly distribution of auxin in the embryo and affect the proper division of embryo cells [43]. The enhancement of pinoid (ENP) is an enhancer of serine/threonine kinase that specifically regulates PIN 1 polarity by cooperating with PID, thereby specifically regulating cotyledon development [44]. In addition, some auxin response factors, such as MNT, which encodes ARF 2, a repressor of cell division regulated by auxin, are also involved in the regulation of seed development and are regulated in response to auxin-related gene expression by the combination of auxin response elements [45]. These genes have a mutated seed size and weight greater than those of the wild type. The GO annotation of the DEGs also revealed that the auxin-activated signaling pathway was significantly enriched, but auxin-related genes related to cottonseed embryo development have not been identified. The germination rate of the cottonseeds after treatment with exogenous IAA (20 mg/L) increased, and further analysis revealed that the levels of sucrose, glucose, and fructose in the cotton seedlings also increased [46]. The expression patterns of 18 genes (*GH_A01G0326*, *GH_A02G0800*, *GH_A02G2059*, *GH_A05G1283*, *GH_A05G1421*, *GH_A05G2265*, *GH_A05G2339*, *GH_A05G4362*, *GH_A08G2175*, *GH_A11G1370*, *GH_A12G2926*, *GH_D01G0311*, *GH_ D02G0815*, *GH_D03G0690*, *GH_D07G1635*, *GH_D07G1893*, *GH_D11G1405* and *GH_D12G2950*) and the key genes of the auxin pathway in embryonic development were altered, and the fold changes in these genes exceeded 10. These auxin-related DEGs can be used as key genes for the study of cotton embryo development in the future, and the specific role of auxin in the development of cotton embryos can be further elucidated through the study of these genes. We identified many pathways involved in glucose metabolism that were significantly enriched. Glyceraldehyde-3-phosphate dehydrogenase (GAPDH), a key enzyme involved in glycolysis and gluconeogenesis, was differentially expressed between and within the lines, indicating that these four GAPDH genes (*GH_A03G1495*, *GH_A05G3573*, *GH_A11G0670* and *GH_D04G0820*) may be crucial for the development of cottonseed embryos.

MADS family genes play a key role in plant growth and development and stress response. Molecular marker detection has revealed that the MADS-box gene *VvAGL11* was a key gene controlling seed abortion [47]. Based on the combined analysis of the transcriptome and genome resequencing of seedless grape plants, two differentially expressed genes, namely, *VvMADS28* and *VvMADS39*, were identified and isolated, and functional verification has revealed that *VvMADS28* is involved in seed development and that *VvMADS39* is associated with grape seedlessness [48]. We identified one candidate gene, *GH_D02G1381*, encoding a MADS transcription factor, and its homologous gene in Arabidopsis is involved mainly in the control of flowering time. In crops such as corn and tomato, RNAi silencing or the overexpression of key enzymes involved in glucose metabolism (acid invertase, sucrose synthase, etc.) has been found to lead to seed abortion or increased seed weight. Our DEGs also annotated many pathways related to glucose metabolism, and there was a candidate gene, *GH_D06G0373*, encoding trehalose 6-phosphate phosphatase, which is involved mainly in starch and sucrose metabolism. The development of seed embryos is related to important agronomic traits such as crop yield and quality, so the study of the mechanism of seed embryo abortion, especially the exploration of genetic and molecular mechanisms, is particularly crucial. At present, the mechanism of seed embryo abortion is still unclear. We screened nine candidate genes related to cotton embryo development via WGCNA, including three TFs. These genes can be used as potential regulatory mechanisms and candidate genes for cotton seed development and molecular breeding in the future. Therefore, with the development of molecular biology, the continuous improvement of whole-genome sequencing, and the improvement of sequencing technology, future research on seed embryo abortion should focus on the cloning of more genes related to abortion in the seed embryo, the clarification of the regulatory pathway and interactions between these genes, and the comprehensive analysis of the mechanism of seed embryo abortion in cotton in combination with physiology, biochemistry, and embryology. In addition, the gene regulatory network involved in the development of cotton embryos is complex. This study analyzed only RNA-seq data collected during the critical period of embryo development and identified several key metabolic pathways. The results have certain limitations. Numerous related metabolites and genes that control seed growth and development should be comprehensively analyzed and explored in the future, and the molecular mechanism and regulatory network of cotton differences should be revealed through the study of the regulatory mechanism of material metabolism during embryo development.

## 5. Conclusions

In this study, we harnessed RNA-seq data across four critical time points to establish a reliable dataset for exploring cottonseed embryo development in a natural mutant and wild-type line. Our investigation not only pinpointed the pivotal stage of seed embryo maturation post 20 DPA but also elucidated key regulatory pathways governing seed embryo development through the identification of DEGs and TFs. The clustering of DEGs facilitated an insightful portrayal of different seed embryo development phases. Furthermore, the exploration using WGCNA led to the identification of nine candidate genes associated with cottonseed embryo development, including three TFs. Despite this progress, further research is required to uncover the precise roles of these genes in *G. hirsutum* seed embryo development. These findings lay the groundwork for a comprehensive understanding of the molecular mechanisms driving cottonseed embryo development while offering a novel genetic repository for future cottonseed embryo investigations.

## Figures and Tables

**Table 1:** Functional annotation of candidate genes.

Gene ID	Gene Name	Functional Annotation
GH_A07G0877	UAM	Amino sugar and nucleotide sugar metabolism
GH_D01G1934	FMOs	Carotenoid biosynthesis
GH_D02G0314	GST	Glutathione metabolism
GH_D02G1381	MADS	Involved in control of flowering time
GH_D03G0690	MYB	Involved in trichome and endosperm development
GH_D06G0373	T6P	Starch and sucrose metabolism
GH_D10G0072	BBE-like	Phenylpropanoid biosynthesis
GH_D10G0604	C3H	Involved in seed germination, seedling/seed development
GH_D11G3021	NOX	MAPK signaling pathway

## Data Availability

The RNA-seq data presented in the study are deposited in the NCBI repository under accession number PRJNA1121102.

## Supplementary Materials

The following supporting information can be downloaded at: https://www.mdpi.com/article/10.3390/genes15111408/s1, Table S1. All the primers used in this study. Table S2. Summary statistics for the RNA-seq analysis of cottonseed embryo samples from *G. hirsutum*.
